# Crystal structure of (*E*)-hex-2-enoic acid

**DOI:** 10.1107/S2056989015007380

**Published:** 2015-04-18

**Authors:** Tim Peppel, Marcel Sonneck, Anke Spannenberg, Sebastian Wohlrab

**Affiliations:** a Leibniz-Institut für Katalyse e. V. an der Universität Rostock, Albert-Einstein-Strasse 29a, 18059 Rostock, Germany

**Keywords:** crystal structure, hydrogen bond, dimer, unsaturated carb­oxy­lic acid

## Abstract

The crystal structure of the title compound, C_6_H_10_O_2_, an α,β-unsaturated carb­oxy­lic acid, displays carb­oxy­lic acid inversion dimers linked by pairs of O—H⋯O hydrogen bonds. The packing is characterized by layers of acid dimers. All the non-H atoms of the (*E*)-hex-2-enoic acid mol­ecule lie almost in the same plane (r.m.s. deviation for the non-H atoms = 0.018 Å).

## Related literature   

For the synthesis of unsaturated α,β-carb­oxy­lic acids including the title compound, see: Shabtai *et al.* (1981 ▸); Lee *et al.* (1990 ▸); Zhang *et al.* (2010 ▸). For crystal structure determinations of related unsaturated carb­oxy­lic acids, see, for acrylic acid: Higgs *et al.* (1963 ▸); Chatani *et al.* (1963 ▸); Boese *et al.* (1999 ▸); Oswald *et al.* (2011 ▸); see, for crotonic acid: Shimizu *et al.* (1974 ▸). For the structures of co-crystals containing the title compound, see: Aakeröy *et al.* (2003 ▸); Stanton & Bak (2008 ▸).
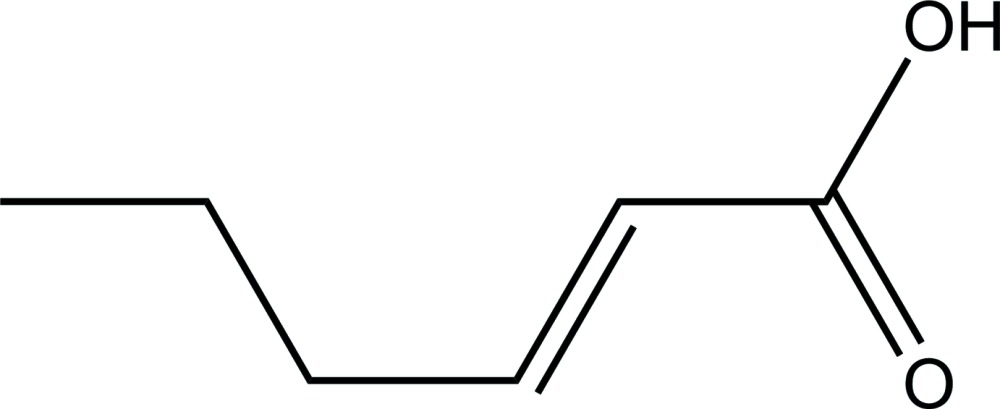


## Experimental   

### Crystal data  

C_6_H_10_O_2_
*M* *_r_* = 114.14
Triclinic, 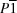

*a* = 6.8556 (3) Å
*b* = 6.9894 (3) Å
*c* = 7.4967 (3) Å
α = 79.477 (1)°
β = 80.620 (1)°
γ = 63.654 (1)°
*V* = 315.12 (2) Å^3^
*Z* = 2
Mo *K*α radiation
μ = 0.09 mm^−1^
*T* = 150 K
0.45 × 0.41 × 0.31 mm

### Data collection  

Bruker APEXII CCD diffractometer
Absorption correction: multi-scan (*SADABS*; Bruker, 2014 ▸) *T*_min_ = 0.91, *T*_max_ = 0.97
5046 measured reflections
1518 independent reflections
1399 reflections with *I* > 2σ(*I*)
*R*_int_ = 0.013

### Refinement  

*R*[*F* ^2^ > 2σ(*F* ^2^)] = 0.034
*wR*(*F* ^2^) = 0.092
*S* = 1.09
1518 reflections
78 parameters
H atoms treated by a mixture of independent and constrained refinement
Δρ_max_ = 0.34 e Å^−3^
Δρ_min_ = −0.16 e Å^−3^

### 

Data collection: *APEX2* (Bruker, 2014 ▸); cell refinement: *SAINT* (Bruker, 2013 ▸); data reduction: *SAINT*; program(s) used to solve structure: *SHELXS97* (Sheldrick, 2008 ▸); program(s) used to refine structure: *SHELXL2014* (Sheldrick, 2015 ▸); molecular graphics: *SHELXL2014*; software used to prepare material for publication: *SHELXL2014*.

## Supplementary Material

Crystal structure: contains datablock(s) I, New_Global_Publ_Block. DOI: 10.1107/S2056989015007380/hb7405sup1.cif

Structure factors: contains datablock(s) I. DOI: 10.1107/S2056989015007380/hb7405Isup2.hkl

Supporting information file. DOI: 10.1107/S2056989015007380/hb7405Isup3.cml

**Fig. 1.**
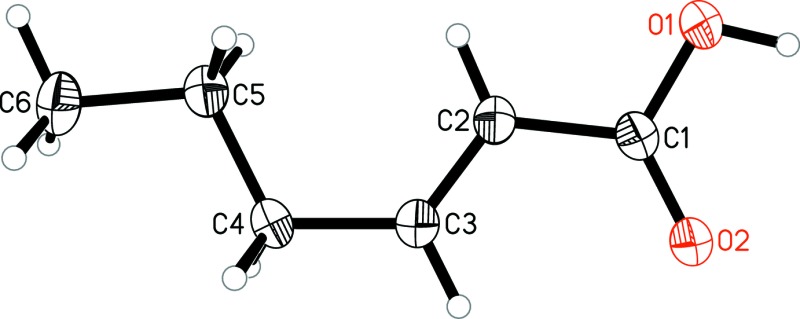
Molecular structure of the title compound with displacement ellipsoids drawn at 50% probability level.

. DOI: 10.1107/S2056989015007380/hb7405fig1.tif
Mol­ecular structure of the title compound with displacement ellipsoids drawn at 50% probability level.

**Fig. 2.**
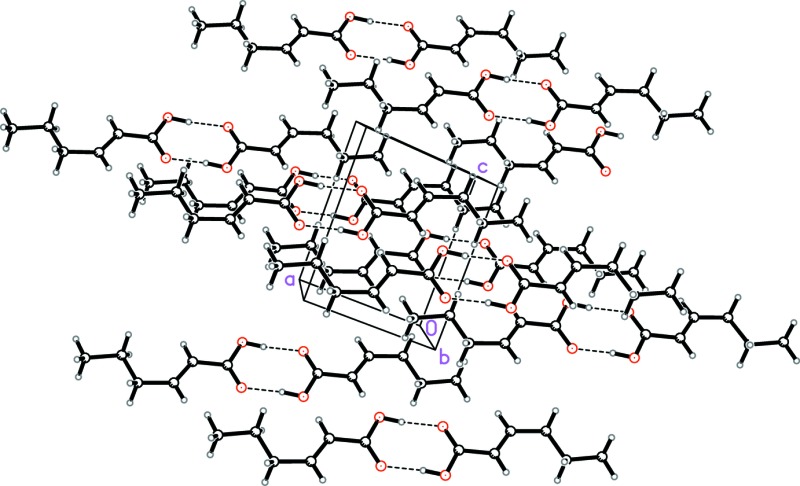
Packing diagram showing O—H···O hydrogen bonding.

. DOI: 10.1107/S2056989015007380/hb7405fig2.tif
Packing diagram showing O—H⋯O hydrogen bonding.

CCDC reference: 1059596

Additional supporting information:  crystallographic information; 3D view; checkCIF report

## Figures and Tables

**Table 1:** Hydrogen-bond geometry (, )

*D*H*A*	*D*H	H*A*	*D* *A*	*D*H*A*
O1H1O2^i^	0.918(19)	1.721(19)	2.6343(9)	173.3(17)

Symmetry code: (i) 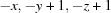.

## supplementary crystallographic information

### S1. Synthesis and crystallization

Malonic acid (24.8 g, 237.8 mmol, 1 eq) was dissolved in dry pyridine (37.6 g, 475.7 mmol, 2 eq) at room temperature in a three-necked flask equipped with a magnetic stir bar and a reflux condenser under a mild flow of argon. Butyraldehyde (17.2 g, 237.8 mmol, 1 eq) was then added in one portion and the resulting clear solution was further stirred for 72 h at room temperature under argon. Afterwards, the resulting light yellow to orange solution was brought to an acidic pH value by adding phospho­ric acid at 0°C (42.5 wt.-%, 582.7 mmol, 2.45 eq). The resulting two layers were extracted three times with 150 ml portions of ethyl acetate and reduced to a volume of ca. 150 ml. To remove impurities from the aldol condensation the raw acid was converted into the corresponding sodium salt by addition of an aqueous solution of sodium carbonate (18.9 g, 178.4 mmol, 0.75 eq in 200 ml). After stirring for 30 minutes the water phase was separated und extracted three times with 150 ml portions of ethyl acetate. The water phase was then acidified with concentrated hydro­chloric acid (35.2 g, 356.7 mmol, 1.5 eq), the organic phase was separated and the water phase was again extracted three times with 150 ml portions of ethyl acetate. The combined organic phases were dried over Na_2_SO_4_ and evaporated to dryness under diminished pressure. The resulting raw product was further purified by distillation *in vacuo* yielding the product in purity >99% (GC). mp. 32°C. ^1^H NMR (400MHz, CDCl_3_): *δ* = 12.13 (br s, 1H, OH); 7.08 (dt, ^3^*J* = 15.6 Hz, ^3^*J* = 7.0 Hz, 1H, -CH-); 5.82 (dt, ^3^*J* = 15.6 Hz, ^4^*J* = 1.6 Hz, 1H, -CH-); 2.23-2.17 (m, 2H, -CH_2_-); 1.49 (ps-sext, *J* = 7.4 Hz, 2H, -CH_2_-); 0.93 (t, ^3^*J* = 7.6 Hz; 3H, -CH_3_-). ^13^C NMR (100MHz, CDCl_3_): *δ* = 172.59 (CO); 152.33 (CH); 120.95 (CH); 34.40 (CH_2_); 21.25 (CH_2_); 13.72 (CH_3_). MS (EI, 70eV): *m/z* = 114 (M^+^, 10), 99 (27), 81 (11), 73 (70), 71 (12), 69 (16), 68 (52), 67 (14), 57 (11), 55 (43), 53 (28), 51 (13), 50 (11), 45 (53), 43 (24), 42 (47), 41 (64), 40 (24), 39 (100), 38 (20), 29 (44). HRMS (ESI-TOF/MS): calculated for C_6_H_10_O_2_ (M^+^) 114.06753, found 114.06768. Elemental analysis for C_6_H_10_O_2_ % (calc.): C 63.13 (63.14); H 8.84 (8.83). Colourless prisms were grown by slow evaporation of an ethano­lic solution at -30 °C over one week.

### S2. Refinement

H1 could be found from the difference Fourier map and was refined freely. All other H atoms were placed in idealized positions with d(C—H) = 0.95 Å (CH), 0.99 Å (CH_2_), 0.98 Å (CH_3_) and refined using a riding model with *U*_iso_(H) fixed at 1.2 *U*_eq_(C) for CH and CH_2_ and 1.5 *U*_eq_(C) for CH_3_.

### Crystal data

**Table:**

C_6_H_10_O_2_	*Z* = 2
*M**_r_* = 114.14	*F*(000) = 124
Triclinic, *P*1	*D*_x_ = 1.203 Mg m^−^^3^
*a* = 6.8556 (3) Å	Mo *K*α radiation, λ = 0.71073 Å
*b* = 6.9894 (3) Å	Cell parameters from 3906 reflections
*c* = 7.4967 (3) Å	θ = 2.8–28.9°
α = 79.477 (1)°	µ = 0.09 mm^−^^1^
β = 80.620 (1)°	*T* = 150 K
γ = 63.654 (1)°	Prism, colourless
*V* = 315.12 (2) Å^3^	0.45 × 0.41 × 0.31 mm

### Data collection

**Table:**

Bruker APEXII CCD diffractometer	1518 independent reflections
Radiation source: fine-focus sealed tube	1399 reflections with *I* > 2σ(*I*)
Detector resolution: 8.3333 pixels mm^-1^	*R*_int_ = 0.013
φ and ω scans	θ_max_ = 28.0°, θ_min_ = 2.8°
Absorption correction: multi-scan (*SADABS*; Bruker, 2014)	*h* = −9→8
*T*_min_ = 0.91, *T*_max_ = 0.97	*k* = −9→9
5046 measured reflections	*l* = −9→9

### Refinement

**Table:**

Refinement on *F*^2^	0 restraints
Least-squares matrix: full	Hydrogen site location: mixed
*R*[*F*^2^ > 2σ(*F*^2^)] = 0.034	H atoms treated by a mixture of independent and constrained refinement
*wR*(*F*^2^) = 0.092	*w* = 1/[σ^2^(*F*_o_^2^) + (0.0441*P*)^2^ + 0.0656*P*] where *P* = (*F*_o_^2^ + 2*F*_c_^2^)/3
*S* = 1.09	(Δ/σ)_max_ < 0.001
1518 reflections	Δρ_max_ = 0.34 e Å^−^^3^
78 parameters	Δρ_min_ = −0.16 e Å^−^^3^

### Special details

**Table:**

Geometry. All e.s.d.'s (except the e.s.d. in the dihedral angle between two l.s. planes) are estimated using the full covariance matrix. The cell e.s.d.'s are taken into account individually in the estimation of e.s.d.'s in distances, angles and torsion angles; correlations between e.s.d.'s in cell parameters are only used when they are defined by crystal symmetry. An approximate (isotropic) treatment of cell e.s.d.'s is used for estimating e.s.d.'s involving l.s. planes.

### Fractional atomic coordinates and isotropic or equivalent isotropic displacement parameters (Å^2^)

**Table:**

	*x*	*y*	*z*	*U*_iso_*/*U*_eq_	
O1	0.25416 (11)	0.27334 (11)	0.55896 (10)	0.03153 (19)	
H1	0.105 (3)	0.340 (3)	0.585 (2)	0.065 (5)*	
O2	0.16993 (10)	0.55854 (11)	0.34602 (9)	0.02998 (19)	
C1	0.30580 (14)	0.39036 (14)	0.41827 (12)	0.0232 (2)	
C2	0.54014 (14)	0.29972 (14)	0.35596 (12)	0.0245 (2)	
H2	0.6366	0.1656	0.4144	0.029*	
C3	0.61808 (14)	0.40324 (14)	0.21920 (12)	0.0246 (2)	
H3	0.5155	0.5375	0.1661	0.030*	
C4	0.84965 (14)	0.33135 (15)	0.13986 (12)	0.0261 (2)	
H4A	0.8960	0.4454	0.1455	0.031*	
H4B	0.8554	0.3186	0.0095	0.031*	
C5	1.01424 (15)	0.12084 (15)	0.22844 (13)	0.0286 (2)	
H5A	0.9700	0.0049	0.2245	0.034*	
H5B	1.0148	0.1327	0.3579	0.034*	
C6	1.24357 (16)	0.06180 (18)	0.13358 (15)	0.0354 (2)	
H6A	1.2465	0.0372	0.0084	0.053*	
H6B	1.3469	−0.0695	0.1996	0.053*	
H6C	1.2850	0.1796	0.1316	0.053*	

### Atomic displacement parameters (Å^2^)

**Table:**

	*U*^11^	*U*^22^	*U*^33^	*U*^12^	*U*^13^	*U*^23^
O1	0.0231 (3)	0.0305 (4)	0.0339 (4)	−0.0104 (3)	0.0043 (3)	0.0036 (3)
O2	0.0214 (3)	0.0309 (4)	0.0305 (4)	−0.0086 (3)	0.0022 (3)	0.0022 (3)
C1	0.0228 (4)	0.0254 (4)	0.0228 (4)	−0.0121 (3)	0.0011 (3)	−0.0045 (3)
C2	0.0201 (4)	0.0247 (4)	0.0266 (4)	−0.0086 (3)	0.0007 (3)	−0.0033 (3)
C3	0.0215 (4)	0.0254 (4)	0.0252 (4)	−0.0091 (3)	−0.0004 (3)	−0.0032 (3)
C4	0.0229 (4)	0.0295 (4)	0.0253 (4)	−0.0128 (4)	0.0030 (3)	−0.0021 (3)
C5	0.0229 (4)	0.0294 (5)	0.0310 (5)	−0.0106 (4)	0.0015 (3)	−0.0029 (4)
C6	0.0222 (4)	0.0390 (5)	0.0417 (6)	−0.0102 (4)	0.0029 (4)	−0.0102 (4)

### Geometric parameters (Å, º)

**Table:**

O1—C1	1.3141 (11)	C4—H4A	0.9900
O1—H1	0.918 (19)	C4—H4B	0.9900
O2—C1	1.2259 (11)	C5—C6	1.5214 (13)
C1—C2	1.4699 (12)	C5—H5A	0.9900
C2—C3	1.3243 (13)	C5—H5B	0.9900
C2—H2	0.9500	C6—H6A	0.9800
C3—C4	1.4900 (12)	C6—H6B	0.9800
C3—H3	0.9500	C6—H6C	0.9800
C4—C5	1.5142 (13)		
			
C1—O1—H1	107.1 (11)	C5—C4—H4B	108.2
O2—C1—O1	122.73 (8)	H4A—C4—H4B	107.3
O2—C1—C2	123.58 (8)	C4—C5—C6	111.81 (8)
O1—C1—C2	113.69 (8)	C4—C5—H5A	109.3
C3—C2—C1	120.65 (8)	C6—C5—H5A	109.3
C3—C2—H2	119.7	C4—C5—H5B	109.3
C1—C2—H2	119.7	C6—C5—H5B	109.3
C2—C3—C4	126.86 (8)	H5A—C5—H5B	107.9
C2—C3—H3	116.6	C5—C6—H6A	109.5
C4—C3—H3	116.6	C5—C6—H6B	109.5
C3—C4—C5	116.56 (7)	H6A—C6—H6B	109.5
C3—C4—H4A	108.2	C5—C6—H6C	109.5
C5—C4—H4A	108.2	H6A—C6—H6C	109.5
C3—C4—H4B	108.2	H6B—C6—H6C	109.5
			
O2—C1—C2—C3	2.65 (14)	C2—C3—C4—C5	−1.89 (14)
O1—C1—C2—C3	−177.75 (8)	C3—C4—C5—C6	178.77 (8)
C1—C2—C3—C4	−179.32 (8)		

### Hydrogen-bond geometry (Å, º)

**Table:**

*D*—H···*A*	*D*—H	H···*A*	*D*···*A*	*D*—H···*A*
O1—H1···O2^i^	0.918 (19)	1.721 (19)	2.6343 (9)	173.3 (17)

Symmetry code: (i) −*x*, −*y*+1, −*z*+1.
