# Supplementary figures and images for: Crystal structure of (E)-hex-2-enoic acid
Source: Acta Crystallogr E Crystallogr Commun. 2015 Apr 18;71(Pt 5):o323. doi: 10.1107/S2056989015007380 (PMC4420068; doi:10.1107/S2056989015007380)

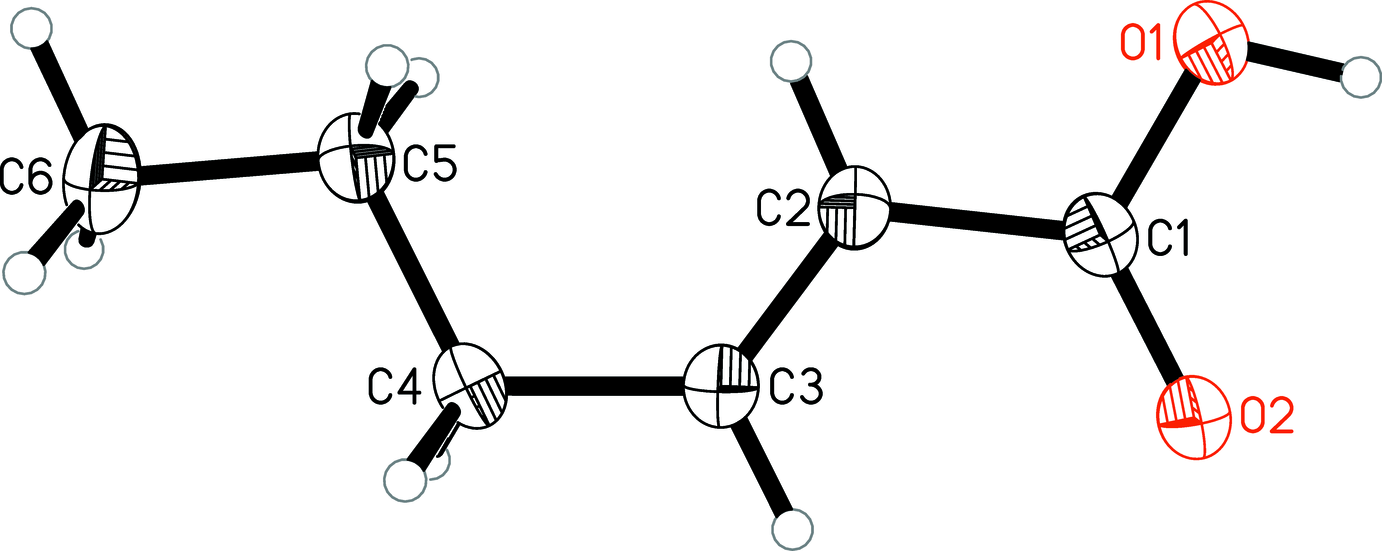

Supplement: Supplementary file 4 [file e-71-0o323-fig1.tif]

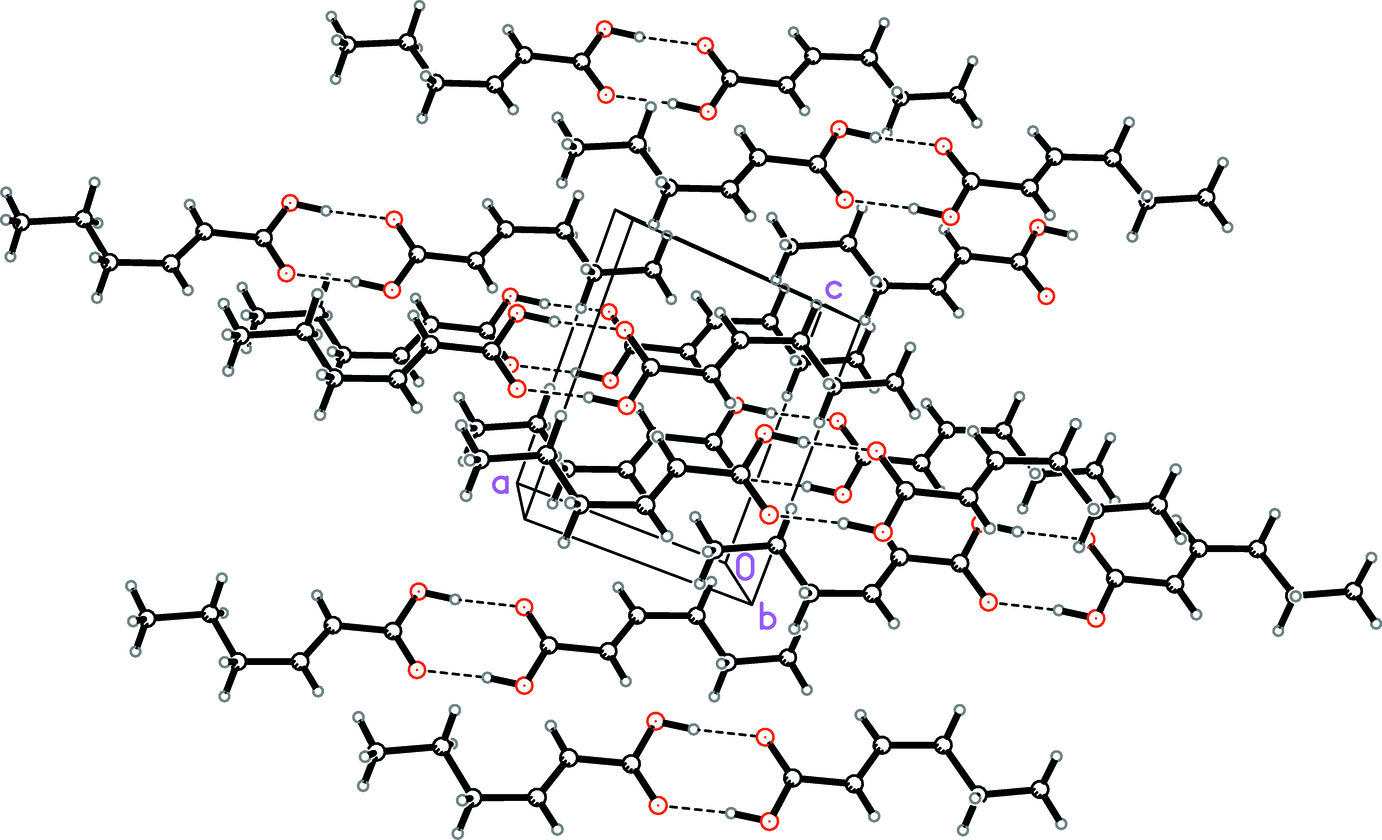

Supplement: Supplementary file 5 [file e-71-0o323-fig2.tif]
